# Prediction of peptides observable by mass spectrometry applied at the experimental set level

**DOI:** 10.1186/1471-2105-8-S7-S23

**Published:** 2007-11-01

**Authors:** William S Sanders, Susan M Bridges, Fiona M McCarthy, Bindu Nanduri, Shane C Burgess

**Affiliations:** 1 Department of Biochemistry & Molecular Biology, Mississippi State University, MS, USA; 2 Department of Computer Science & Engineering, Mississippi State University, MS, USA; 3 Department of Basic Sciences, College of Veterinary Medicine, Mississippi State University, MS, USA; 4 Institute for Digital Biology, Mississippi State University, MS, USA; 5 Mississippi Agriculture and Forestry Experiment Station, Mississippi State University, MS, USA

## Abstract

**Background:**

When proteins are subjected to proteolytic digestion and analyzed by mass spectrometry using a method such as 2D LC MS/MS, only a portion of the proteotypic peptides associated with each protein will be observed. The ability to predict which peptides can and cannot potentially be observed for a particular experimental dataset has several important applications in proteomics research including calculation of peptide coverage in terms of potentially detectable peptides, systems biology analysis of data sets, and protein quantification.

**Results:**

We have developed a methodology for constructing artificial neural networks that can be used to predict which peptides are potentially observable for a given set of experimental, instrumental, and analytical conditions for 2D LC MS/MS (a.k.a Multidimensional Protein Identification Technology [MudPIT]) datasets. Neural network classifiers constructed using this procedure for two MudPIT datasets exhibit 10-fold cross validation accuracy of about 80%. We show that a classifier constructed for one dataset has poor predictive performance with the other dataset, thus demonstrating the need for dataset specific classifiers. Classification results with each dataset are used to compute informative percent amino acid coverage statistics for each protein in terms of the predicted detectable peptides in addition to the percent coverage of the complete sequence. We also demonstrate the utility of predicted peptide observability for systems analysis to help determine if proteins that were expected but not observed generate sufficient peptides for detection.

**Conclusion:**

Classifiers that accurately predict the likelihood of detecting proteotypic peptides by mass spectrometry provide proteomics researchers with powerful new approaches for data analysis. We demonstrate that the procedure we have developed for building a classifier based on an individual experimental data set results in classifiers with accuracy comparable to those reported in the literature based on large training sets collected from multiple experiments. Our approach allows the researcher to construct a classifier that is specific for the experimental, instrument, and analytical conditions of a single experiment and amenable to local, condition-specific, implementation. The resulting classifiers have application in a number of areas such as determination of peptide coverage for protein identification, pathway analysis, and protein quantification.

## Background

In high-throughput non-electrophoretic proteomics complex mixtures of proteins are subjected to proteolytic digestion with an enzyme such as trypsin before the fragments are separated by liquid chromatography (LC) and analyzed by tandem mass spectrometry. However, for a particular protein, only a portion of the peptides are actually observed experimentally and the set of peptides that are observed from a single protein can vary substantially from one experiment to another. A number of factors contribute to the inability to detect some peptides and to variations in the peptides that are detected from one experiment to another. These include incomplete proteolytic digestion, small size, poor binding or elution from the type of LC column used, the limited mass range that can be detected by the mass spectrometer, bias toward detecting peptides with an intense MS signal in mixtures, the phenomenon of "ion suppression", the charge prior to ionization, and non-covalent interactions between peptides in the gas phase while in the mass spectrometer [1]. In addition, are substantial differences in the peptides observed due to variations in the protein extraction and or solublization methods, tissue types, prefractionation, LC separation conditions, and differences between gradients even when the same LC separation conditions are used. Furthermore, different databases, different search software and even different versions of the same software also influence which peptides that are detected.

We refer to peptides that can be detected as "flyable". The fact that most proteins in a complex mixture are represented by only a small number of proteolytic peptides presents several difficulties for proteomics researchers [2]. These problems include assessment of the level of confidence in protein identifications [3], determining the peptide coverage of proteins [4], determining if "missing" proteins are potentially observable [5,6], and using peptide observability as an adjustment factor for protein quantification based on observed peptides [4,7]. Recently reported methods for predicting peptide observability have been based on large training datasets from multiple experiments dealing with a single organism [4,7]. However, because the observability of peptides depends not only on the properties of the peptides themselves but also on specific experimental, instrumental, and analytical procedures, we contend that it is necessary to provide a method for predicting peptide observability for a specific experimental set at the local level. This ability to construct a classifier for a particular dataset is particularly important for researchers who work in smaller laboratories, deal with a variety of organisms and/or tissues, employ a variety of protein extraction protocols, and/or who use a centralized facility for proteomics where they have little control over instrumental and analytical protocols.

Here we describe a method for constructing a classifier for a proteomics data set that can predict peptide observability for a particular set of experimental conditions. We demonstrate that the classifiers constructed using this method provide critical information for assessing the validity of protein identifications and valuable evidence to support competing hypotheses about the presence or absence of "missing" proteins in a pathway of interest.

The set of tryptic peptides that are observed under experimental conditions can be divided into two classes – proteotrypic and flyable. Proteotrypic peptides are those experimentally observable peptides that can be used to uniquely identify a protein, while flyable peptides are all peptides that are experimentally observable but may not be proteotrypic [8]. Proteotypic peptides are a subset of flyable peptides and flyable peptides are a subset of all possible tryptic peptides. The spectra generated by mass spectrometry analysis of a complex peptide mixture are matched against theoretical spectra generated from an *in silico *trypsin-digested protein database. The resulting set of peptide identifications is then used for protein identification. By definition, detection of at least one proteotypic peptide is required for protein identification.

There is, however, disagreement among researchers about the number of peptide matches and the peptide coverage of the protein that are required for an identification to be considered valid. Protein identifications based on a single proteotypic peptide (sometimes called "one hit wonders") are often viewed with skepticism. Some researchers contend that a protein identification needs at least two proteotypic peptides to be valid, while others contend that a single high quality peptide can be used for identification purposes [3]. Furthermore, some proteins produce only one proteotypic peptide. In addition to the number of peptides identified, the degree of coverage of the protein by peptides may also be used as a measure to assess the validity of the identification – this is typically provided in terms of the percentage of amino acids in the protein "covered" by identified peptides. However, an additional and more meaningful statistic is the percentage of potentially detectable peptides that are observed. This information has the potential to increase (or decrease) the credibility of some single proteotypic peptides for identification and can prevent loss of important data [3] or the inclusion of erroneous identifications.

Researchers using proteomics are interested in not only cataloging proteins present, but also in studying the location and differential expression of the proteins involved in biochemical pathways [2]. Often, one or more proteins referenced to participate in a canonical pathway are not observed in a proteomics dataset, but most other proteins in the pathway are present [5,6]. Conversely, a protein that has never been identified in that pathway may be identified by a single proteotypic peptide. In the first case, it is important to know whether these missing proteins generate a sufficient number of potentially observable proteotypic peptides to support identification under the specific experimental conditions or whether the protein truly appears to be absent. In the second case, it is important to determine if a protein may reasonably be expected to be identified by only one peptide under the experimental conditions – an identification of a protein with a single peptide where the protein is predicted to produce many observable proteotypic peptides should be viewed with suspicion.

Two recently published papers describe methods for the prediction of peptide detection using mass spectrometry, but their methods are distinct from ours. Mallick et al. [4] have compiled a large training set from multiple yeast proteomics experiments and built Gaussian mixture discriminant function predictors for a number of different proteomics platforms. Their goal is to characterize the general properties of peptides that can be detected using different proteomics technologies, to determine the coverage of the predicted proteome that is detectable using different technologies, and they also argue that their method can be used to improve protein quantification. Lu et al. [7] describe a classifier for predicting peptide observability that is a component of a method for absolute protein quantification and that adjusts scores for protein abundance based on the predicted detectability of *in silico *generated tryptic peptides. In contrast, our procedure is specifically developed for generating a classifier for a single data set to predict flyable peptides for a particular set of experimental conditions (biological sample, protein extraction protocol, mass spectrometric instrumentation, HPLC column type, database search algorithm and settings, etc.) and to be applied locally. We demonstrate that the resulting classification provides valuable information with regard to peptide coverage of a protein and can assist the proteomics researcher in a systems analysis of the dataset.

## Results and discussion

**Figure 1:**
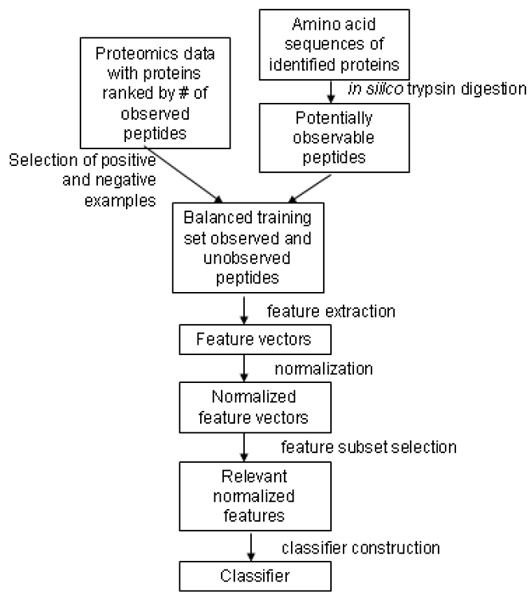
Classifier construction process.

We have developed a procedure for building a classifier to predict peptide flyability from a proteomics dataset. The output of the protein identification algorithms for a proteomics dataset includes the proteins that were identified and the peptides that were used for each protein identification. As Figure 1 illustrates, the classifier construction process includes selection of a set of observed and unobserved peptides for the training set, extraction of features to represent the peptides in the training set, normalization of the feature values, feature subset selection, and training and testing of the classifier.

### Training set compilation strategy

The first step in the process is selection of a set of peptides for the training data set. The naïve approach is to use all observed peptides for the positive examples and all non-observed *in silico *generated peptides from identified proteins for the negative examples. However, this approach ignores several complications that arise when processing proteomics datasets. First, some of the "observed" peptides will be false positive identifications. The probability that a peptide is a false positive identification is greatly reduced if it is one of multiple peptides used to identify a protein since the probability of this occurring by chance is small [3]. Therefore, we limit the positive examples to the peptides associated with proteins that were identified using multiple unique peptides. Peptides chosen for negative examples are also limited to the set of proteins identified by multiple peptides. However, selection of negative examples is also complicated by the fact that the number peptides observed for a protein is directly related to protein abundance in the sample. Isotope-free quantification methods for proteomics datasets make use of the relationship between the number of peptides observed and protein concentration [9-12]. To avoid the problem of labeling peptides that were not observed as negative examples because they are associated with low abundance proteins, we have chosen to compile the negative examples from the proteins that were identified with the largest number of peptides. Although this introduces a bias for peptides from abundant and large proteins, this strategy insures, to the extent possible, that the peptides used for negative examples were present in sufficient quantity to be potentially observable. We have developed the following procedure for selection of the training set to ensure that the peptides selected for the class of observable peptides are high confidence identifications and that the peptides selected for the negative examples are truly "unobservable" under the specific experimental conditions.

1. Rank the protein identifications by the number of peptides used in the identification and include only identifications based at least two distinct peptides.

2. Retrieve the amino acid sequence for each of the proteins in step 1, perform *in silico *trypsin (or appropriate enzyme) digestion of the proteins, and compile a list of all predicted tryptic peptides of length greater than 6 amino acids (because this number gives a probability of the sequence identifying another sequence at random of 1 in 19^6 ^and which is reasonable for a eukaryote genome of around 4 billion base-pairs such as human).

3. If a peptide is present in the experimental data, it is assigned a value of 1 and if it is not observed in the experimental data it is given a value of 0. There will be many more with a value of 0 than with 1.

4. The peptides labeled with a 1 in the previous step are used as the positive examples in the training set. Suppose the size of this set is *n*. In order assure that peptides used as negative examples were present in sufficient quantity for detection and to also help produce a balanced training set, we select the first *n *"unobserved" peptides from the proteins ranked by the number of peptides used for identification.

### Feature generation and classifier construction

Our approach for generating features to represent each peptide in the training set uses both the features listed in Table 1 (called Feature Set 1) and features constructed using properties from the AAIndex [13]. The first set of features (see Table 1) includes basic properties of the peptide (e.g. mass and size) and features related to the amino acid composition of the peptide. The AA Index is a compilation in a set of tables of 544 different indices used to characterize amino acids. It includes indices for wide variety of characteristics of amino acids including hydrophobicity, participation in certain types of structures, etc. A feature value was generated for each peptide for each index representing the sum of the index values for all amino acids in the peptide. Combination of Feature Set 1 and the AAindex features results in a total of 596 features for each peptide. Although this set includes a large number of redundant features, we have shown that using both sets as input for the feature selection process yields improved classifier performance over use of each feature set alone. For example, with the avian bursal dataset described below, the 10-fold classification accuracy of neural networks built with the AAIndex features only is 72%, with Feature Set 1 only is 71%, and with both feature sets is 81%. Because the values of the features cover a wide range of numeric values, NV normalization is used to make the numeric range of all features 0–1. Feature subset selection is then performed to find the set of feature most relevant to the task of predicting flyability and to remove redundant and non-informative features. We use a feature selection method that performs a greedy search through feature space to identify features based on the level of consistency with class values when the training data is compared to the entire set of attributes [14]. The reduced set of features is used to train the classifier. A 3-layer neural network classifier is constructed with an input unit for each of the selected features, *(i+1)/2 *hidden units where *i *is the number of input units, and a single output unit. The neural network is trained using the training set constructed with the strategy described above and tested using 10-fold cross validation. Multilayer neural networks provide a robust method for learning a functional mapping from numeric attribute values to a class value – in this case a mapping from numeric features describing the peptide to the classes "observable" and "unobservable."

**Table 1:** A list of initial features used for classifier construction in addition to AAIndex features.

**Feature Subset 1**
Length of peptide
Net charge of peptide
Positive charge
Negative charge
Isoelectric point
Molecular weight
Hydropathicity
Count of each amino acid (20 features)
Percent composition of each amino acid (20 features)
Percent polar amino acids
Percent positive amino acids
Percent negative amino acids
Percent hydrophobic amino acids

A feature selection procedure is used to reduce dimensionality prior to classifier construction.

In order to demonstrate the utility of our approach, we have used the methodology described above to build classifiers for two different published MudPIT data sets: 1) an avian bursa of Fabricius data set consisting of 5198 proteins [6], and 2) a Hodgkin's lymphoma model data set consisting of 3983 proteins [5]. The classifiers built using our procedure had 10-fold cross validation classification accuracies of 81% and 72% respectively. Table 2 lists the features selected that best distinguish observed peptides from unobservable peptides for both datasets. Table 3 reports the accuracy and confusion matrices for the neural networks for both data sets based on 10-fold cross validation.

**Table 2:** Description of features selected for the classifiers built for the two datasets.

Avian Bursa Dataset
Number of prolines
Percent glycine
Percent alanine
Percent leucine
Percent polar amino acids
Percent hydrophobic amino acids
Percent positive amino acids
Percent negative
Size (Dawson, 1972)
Optimized transfer energy parameter (Oobatake et al., 1985)
Weights for beta-sheet at the window position of 5 (Qian-Sejnowski, 1988)
Transfer free energy from oct to wat (Radzicka-Wolfenden, 1988)
Information measure for C-terminal turn (Robson-Suzuki, 1976)
Amphiphilicity index (Mitaku et al., 2002)

Hodgkin's Lymphoma Model Dataset

Number of cytosienes
Signal sequence helical potential (Argos et al., 1982)
Transer free energy to surface (Bull-Breese, 1974)
Normalized relative frequency of alpha-helix (Isogai et al., 1980)
Normalized relative frequence of double bend (Isogai et al., 1980)
Distance between C-alpha and centroid fo side chain (Levitt, 1976)
Retention coefficient in NAH2PO4 (Meek-Rossetti, 1981)
Interior composition of amino acids intracellular proteins (Fukuchi-Nishikawa, 2001)
Linker propensity from 1-linker dataset (George-Heringa, 2003)

**Table 3:** 10-fold cross-validation accuracy by class for neural networks generated for two datasets.

Class	True positive rate	False positive rate	Precision	Recall	ROC Area
Avian Bursal Dataset					
Not observed	0.80	0.19	0.81	0.80	0.87
Observed	0.82	0.20	0.80	0.82	0.87
					
Hodgkin's Lymphoma Model Dataset					
Not observed	0.66	0.22	0.75	0.66	0.80
Observed	0.78	0.34	0.70	0.78	0.80

The features selected tend to be related to structural properties of the peptides. For example, consider the features selected for the avian bursa classifier. Prolines tend to break alpha helices and prolines located adjacent to lysine or arginine also interfere with trypsin digestion. Amino acids with small side chains such as glycine and alanine increase the flexibility of the peptide. The charge, polarity, hydrophobicity, and the behavior of the peptide in solvent also influence flyability.

Our classifiers achieved classification accuracies comparable to the rates reported by Mallick et al. [4] and Lu et al. [7] for much simpler yeast systems. The accuracy statistics reported by Mallick et al. are difficult to compare to ours because they report specificity in terms of (1 - positive predictive ratio) where the positive predictive ratio is defined as (true positives/(true positives + false positives)) rather than the more traditional true positive ratio (true positives/(true positives + false negatives). Lu et al. report a 69% true positive rate for observed and a 90% true positive rate for non-observed. Note that it is possible to achieve an 82% true positive rate for the non-observed class for their classifier by guessing non-observed in every case. In addition, they include very small peptides (3–5 aa) in their analysis and we exclude peptides of this length from our study because of the high probability of random matches to multiple proteins and their lack of power as unique identifiers.

In order to evaluate the importance of building classifiers that are specific for a particular dataset, we tested each of the classifiers above with the data used for training the other classifier (i.e. avian bursal classifier with Hodgkin's lymphoma model data set as test set and vice versa). The results (Table 4) demonstrate that there is a substantial loss of classifier accuracy when using a classifier trained with one data set to predict peptide observability with the other data set. In both cases, the true positive rate (prediction of observability) decreased dramatically (almost to the level that would be achieved by random guessing). These results are consistent with those reported by Mallick et al. [4] when a classifier trained with yeast data was used to predict observability with human data. These results clearly demonstrate the need for classifiers to be trained for each experimental set.

**Table 4:** Accuracy by class for neural networks generated using one dataset as the training set and the other dataset for test data.

Class	True positive rate	False positive rate	Precision	Recall	ROC Area
Avian Bursal Dataset training set, Hodgkins Lymphoma test set					
Not observed	0.71	0.46	0.61	0.71	0.70
Observed	0.54	0.29	0.66	0.54	0.70
					
Hodgkin's Lymphoma Model Dataset training set, Avian Bursa test set					
Not observed	0.81	0.41	0.81	0.73	0.73
Observed	0.59	0.19	0.59	0.66	0.73

We use the two classifiers described above for the avian bursa dataset and the Hodgkin's lymphoma model dataset to demonstrate the utility of the classifiers for calculating an informative peptide coverage statistic for proteins and for analysis of system's biology datasets. In Table 5, for a subset of proteins that were observed in the data, the total number of tryptic peptides generated by *in silico *tryptic digestion, the number observed, the number of peptides predicted to be detectable by each classifier, and the amino acid coverage both in terms of the total number of tryptic peptides and in terms of those predicted to be observable. As expected, in most cases the amino average coverage for peptides predicted to be detectable is higher, sometimes substantially higher, than the total amino acid coverage. In general, this approach allows the researcher to determine how many peptides might reasonably be expected to be detected.

**Table 5:** Number of tryptic peptides predicted to be observable for selected proteins from the two data sets.

Protein GI Number	Num tryptic peptides (>= 6 aa)	Num tryptic peptides observed	Percent amino acid coverage	Number predicted detectable	Percent predicted detectable	Percent amino acid coverage of detectable
Avian bursa data set						

5902793	20	2	10	9	45	33
119359	50	5	9	15	30	21
128413	16	2	11	3	18	14
2119012	7	2	28	3	43	17
17025728	16	2	6	7	44	20
122000	6	4	33	0	0	0
1762374	7	1	23	2	29	21
1172808	13	1	6	4	30	19
7512219	44	1	2	11	25	34
104697	9	2	22	4	44	30
118106991	12	0	0	0	0	0

Hodgkin's lymphoma model data set						

479367	34	1	3	5	15	11
729629	18	2	14	11	61	43
899264	13	1	10	4	31	21
63544	48	2	2	6	13	15
50750413	38	3	11	8	21	25
45433516	26	0	0	0	0	0
46048702	14	0	0	0	0	0
125745137	9	0	0	0	0	0
125745114	9	0	0	0	0	0
45433516	26	0	0	0	0	0

1) For the avian bursa dataset, 10 randomly selected observed proteins and the DR3 protein that was expected but not observed. 2) For the Hodgkin's lymphoma model dataset, 5 proteins that were observed in the pathway under consideration and 5 that had been observed using other methods in previous experiments but not observed in this dataset.

We have also used the bursal neural network and the Hodgkin's lymphoma model neural network to determine if proteins that are "missing" from a pathway of interest are likely to be potentially observable. The results are given in Table 5. As McCarthy et al. [6] reported, most components of the programmed cell death pathway with known orthologs in chicken were observed in the avian bursa data set with the exception of the protein DR3. The peptides produced by *in silico *tryptic digestion of DR3 (GI 118106991) were used as input to our neural network for this data set. As shown in Table 5, none of the peptides for this protein were predicted to be observable. In contrast, for proteins that were observed, the average number of observable peptides was 5. For the Hodgkin's lymphoma model dataset, there were five proteins that we expected to observe because we have observed them using other methods in other experiments [15,16] but we did not see them in this experiment (also shown in Table 5). The results in Table 5 show that none of the tryptic peptides for these proteins is predicted to be observable under the given experimental conditions while a set of proteins of similar size that were observed were predicted to be observable. Although these results cannot be used to demonstrate conclusively that a protein does or does not exist in a data set, they can be used as one piece of evidence to confirm or refute a hypothesis about the presence of a protein under certain conditions and to plan further wet lab experiments.

## Conclusion

We present a procedure for constructing a classifier to predict which tryptic peptides in a protein are likely to be detectable by mass spectrometry for a specific set of experimental and instrumental conditions. We demonstrate that it is possible to construct a classifier with accuracy comparable to those previously reported based on the accumulation of large training sets from multiple experiments. We also show that a classifier constructed based on one dataset does not perform at an acceptable level when predicting observability for another dataset and thus it is necessary to construct classifiers that are specific for one set of experimental conditions. The resulting classifier provides researchers with a tool that can provide information about peptide coverage of proteins in terms of which proteins are likely to be detectable. It can also be used as one line of evidence in a systems analysis to evaluate alternative hypotheses concerning proteins that were not observed but that were expected. If the "missing" protein generates many predicted detectable peptides but none were observed, then this provides additional probabilistic evidence of absence of the protein – a very difficult hypothesis to demonstrate conclusively. The classifier allows researchers to distinguish between proteins that are not likely to be detected with the methodology versus proteins that were not expressed in the biological system. Only by making this distinction is it possible to accurately interpret proteomics results and improve biological modeling.

## Methods

### Biological samples

Methods used to collect the biological samples, analyze the samples using mass spectrometry, and identify proteins are described in detail in[5] and [6]. All samples were analyzed by MudPIT using an LCQ Deca XP Plus IT mass spectrometer and database search was conducted using TurboSEQUEST (Bioworks Browser; ThermoElectron).

### Software

Custom Perl scripts were written to extract the accessions of proteins and lists of peptides from Sequest output files, to query NCBI and download the protein sequences, to trypsin digest the proteins, to determine which peptides had been observed in the dataset, to select the positive and negative peptides for the data sets, and to compute the feature vectors for each peptide. The software implements the rules for trypsin digestion described for the ExPASy PeptideCutter tool [17]. WEKA Explorer Version 3.4.10, a software package containing a collection of machine learning algorithms for data mining available at [14] was used for feature selection, and building and testing the classifier. The software that generates a training set from a Sequest output file and a detailed readme describing how to generate classifiers for a specific dataset using Weka is available for download in the Tools section of AgBase .

## Competing interests

The authors declare that they have no competing interests.

## Authors' contributions

WSS and SMB developed the method for building the classifier and designed and implemented the software. FMC, BN, and SCB formulated the peptide flyability problem, provided data for developing and testing the system, and assisted with the program design and analysis of results.
